# Robust SERS Platforms Based on Annealed Gold Nanostructures Formed on Ultrafine Glass Substrates for Various (Bio)Applications

**DOI:** 10.3390/bios9020053

**Published:** 2019-04-10

**Authors:** Lan Zhou, Simone Poggesi, Giuliocesare Casari Bariani, Rakesh Mittapalli, Pierre-Michel Adam, Marisa Manzano, Rodica Elena Ionescu

**Affiliations:** 1 Light, Nanomaterials and Nanotechnology (L2N), FRE-CNRS 2019, Institute Charles Delaunay (ICD), University of Technology of Troyes, 12 Rue Marie Curie CS 42060, 10004 Troyes CEDEX, France; lan.zhou@utt.fr (L.Z.); poggesi.simone@spes.uniud.it (S.P.); casaribariani.giuliocesare@spes.uniud.it (G.C.B.); rakesh.mittapalli@utt.fr (R.M.); pierre_michel.adam@utt.fr (P.-M.A.); 2 Dipartimento di Scienze Agroalimentari, Ambientali e Animali (DI4A), Università degli Studi di Udine, Via Sondrio 2/A, 33100 Udine, Italy; marisa.manzano@uniud.it

**Keywords:** SERS on ultrafine solid supports, glass coverslips, BPE, thiol-DNA probe, annealed gold nanostructures

## Abstract

In this study, stable gold nanoparticles (AuNPs) are fabricated for the first time on commercial ultrafine glass coverslips coated with gold thin layers (2 nm, 4 nm, 6 nm, and 8 nm) at 25 °C and annealed at high temperatures (350 °C, 450 °C, and 550 °C) on a hot plate for different periods of time. Such gold nanostructured coverslips were systematically tested via surface enhanced Raman spectroscopy (SERS) to identify their spectral performances in the presence of different concentrations of a model molecule, namely 1,2-bis-(4-pyridyl)-ethene (BPE). By using these SERS platforms, it is possible to detect BPE traces (10^−12^ M) in aqueous solutions in 120 s. The stability of SERS spectra over five weeks of thiol-DNA probe (2 µL) deposited on gold nano-structured coverslip is also reported.

## 1. Introduction

In recent decades, the use of gold nanoparticles (AuNPs) in the field of light–matter interactions has attracted considerable interest for their potential applications in various sciences, such as biomedical, agricultural, environmental, and forensic investigations, because of their unique optical and chemical properties. AuNPs serve as miniaturized platforms, ideal for the development of ultrasensitive bioassays [1,2]. In fact, a considerable number of protocols have been developed for the preparation of AuNPs, which can be classified into three main groups: (i) the top-down approach based on physical manipulation, for example using an ultrasonic field, electron beam lithography [3], or laser irradiation [4]; (ii) the bottom-up method based on the chemical reduction of chloroauric acid to AuNPs in the presence of reducing and stabilizing agents [5], and (iii) the “on solid supports” approach, using an annealed microscope glass slide coated with thin gold film [6,7,8,9,10].

Among the optical methods, surface enhanced Raman spectroscopy (SERS) has attracted scientists’ attention, being used in the identification of unknown substances in analytical chemistry [11], electrochemistry [12], physical chemistry [13], solid state physics, biochemistry, biophysics, and even medicine [14,15,16]. Nowadays, SERS effects on metal (Au)-coated surfaces are explained using electromagnetic and chemical mechanisms [17,18]. The SERS electromagnetic mechanism is caused by the interactions between the laser excitation on the metal-labeled surface and the scattered Raman field. The chemical mechanism is caused by the inelastic tunneling of ballistic electrons to the lowest unoccupied molecular orbital of the chemisorbed molecule. The return of the electron to its initial state in the metal—i.e., the recombination of the electron and the hole—emits a Raman-shifted photon [19,20,21]. Usually, SERS substrates are fabricated either by immobilizing a colloidal silver nanoparticle (AgNP) on 3-aminopropyltriethoxysilane-coated glass coverslips [22] or by dropping tiny volumes of colloidal AgNPs onto microscope glass coverslips [23,24,25]. Despite the simplicity, such SERS substrates are not stable, and AgNPs are easily displayed by water streams. On the contrary, for biological applications, naked AgNPs must be strongly attached to transparent and biocompatible solid supports for further use in different chemical and biomolecule functionalization steps without any nanoparticle displacements. To solve the inconvenience of the stability of nanoparticles on solid supports, a solution was reported in 2013, which consisted of heating the gold-coated microscope glass pieces at a high annealing temperature in an oven for 8 h [6]. However, these substrates require training to carefully cut the microscope slide into small pieces to avoid scratching that may affect the homogeneity in the AgNP formation.

A second solution is proposed in the present work and consists of replacing microscope glass pieces with ultrafine coverslips, thus eliminating the cutting step. It should be noted that glass coverslips are typically used for running conventional biological assays and have never been used for robust SERS (bio)applications. The aim of this work is therefore to validate the use of ultrafine glass coverslips as easy-to-handle and inexpensive SERS supports after a high annealing treatment on a hot plate for several hours. A wide variety of chemicals and biomolecules can be detected with these new SERS platforms. To prove the concept, a Raman model molecule, 1,2-bis-(4-pyridyl)-ethene (BPE) [26,27], was selected to study the SERS spectroscopic performances of annealed gold nanostructures on ultrafine glass coverslips.

## 2. Materials and Methods

### 2.1. Materials

The coverslips were cleaned using Decon 90 (Decon Laboratories^TM^ Decon 90^TM^) liquid detergent (Fisher Scientific, Göteborg, Sweden) and ultrapure water (18.2 MΩ cm) produced by a Millipore Milli-Q water purification system (Molsheim, France). The same water was used for all rinsing steps.

For SERS investigations, a BPE (1,2-bis-(4-pyridyl)-ethene) molecule was purchased from Sigma-Aldrich (Schnelldorf, Germany). Several BPE solutions were prepared from 97% concentrated stock solution, to form six concentrations that were subsequently tested in ultrapure water: 10^−3^, 10^−5^, 10^−7^, 10^−9^, 10^−12^, and 10^−15^ M, respectively. For SERS stability studies, a fragment of DNA modified in 5′ position with C6 thiol group (TGTTTGAGCGTCATTTCCTTCTCACTATTTAGTGGTTATGAGATTACACGAGG, 53 pb), provided by Eurofins Genomics (Eberseberg, Germany) and here called thiol-DNA probe (10 ng/µL), was suspended in 1xSSPE buffer containing 3 M sodium chloride, 0.23 M sodium phosphate dibasic, 25 mM ethylenediaminetetraacetic acid, pH 7.4. The thiol-DNA was designed to detect *Brettanomyces bruxellensis* spoilage yeast. All the reagents required for the preparation of the SSPE buffer were provided by Sigma-Aldrich.

### 2.2. Instruments for the Characterization of Gold Nanoparticles Annealed on Coverslips

Metal evaporation was performed with Plassys MEB 400 (Plassys, Bestek, France). A hot plate (Thermo Fisher Scientific, Waltham, MA, USA) was used for annealing under clean room conditions.

Nanostructured coverslips were characterized with a scanning electron microscope (SEM) (FEG-SU8030, Tokyo, Japan) and an atomic force microscope (AFM) (Bruker ICON, Billeric, MA, USA) with cantilever ScanAsyst-Air in silicon nitride with a tip height of 2.5–8.0 mm. A spring constant of 4 N/m and a reflective aluminum coating on the back side in standard ScanAsyst-Air mode were used to characterize the morphology of AuNPs (data not shown).

SERS spectra were recorded with backscattering geometry using a modified Jobin-Yvon LabRAM (Horiba scientific, Longjumeau, France) and an excitation wavelength of 632.8 nm (11 mW) from the He–Ne laser source, and all the spectra were recorded with a 10× objective Olympus MPlanFl with a 5.2 μm^2^ laser spot area. The acquisition time varied from 10 to 120 s, and all the spectra were recorded 3 times with a D filter range between 0 and 0.3.

For sterilization, a Tuttnauer Autoclave Steam Sterilizer 2540ML (Tuttnauer, Villenoy France) was used. The samples were dried in an oven provided by VWR company (DRY-Line drying oven DL 53), and all operations were made under a biological hood provided by Thermo-scientific MSC 1,2 ADV (Illkirch Cedex, France).

### 2.3. Sample Preparation: Cleaning, Gold Evaporation, and Annealing of Coverslips

**Figure 1:**
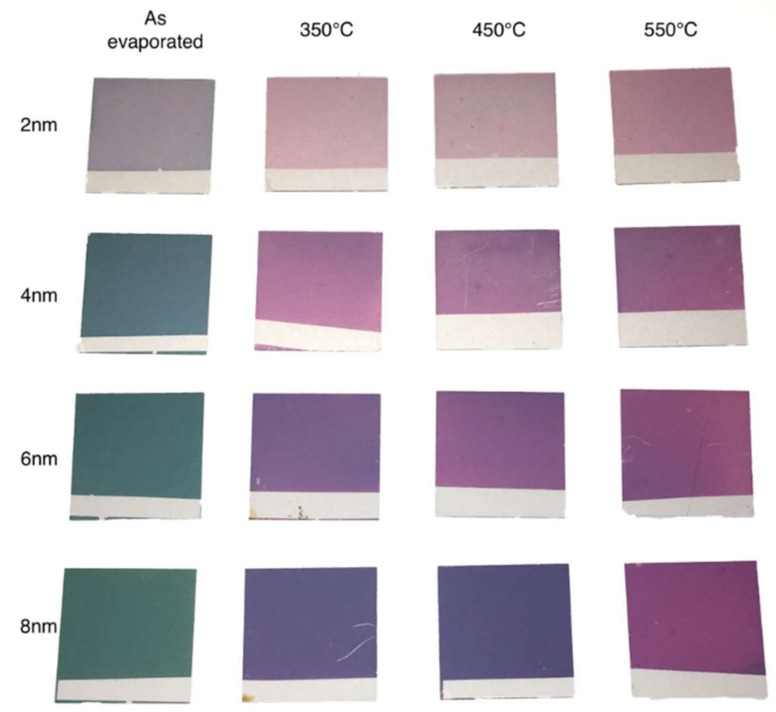
Square glass coverslips coated with gold thin films (2 nm, 4 nm, 6 nm, and 8 nm) after 3 h at three different temperatures (350 °C, 450 °C, and 550 °C).

Glass coverslips (Carl Roth GmbH + Co., KG, Karlsruhe, Germany) were degreased with Millipore distilled water and a detergent solution (Decon 90) (ratio 2:8, *v*/*v*) in an ultrasonic distilled water bath (Elmasonic S30H model, Elma Schmidbauer GmbH, Singen, Germany) at 50 °C for 15 min according to the procedure used by Jia et al. [6]. In addition, an ultrasonic bath was made with distilled water at 50 °C for 5 min. The next step was to carefully rinse each coverslip with distilled water, dry them under a stream of nitrogen, and deposit them on a hot plate at 100 °C for 10 min. Further, the coverslips were labelled with a scotch band on an external side for correct handling, fixed on a circular evaporation plate (200 mm diameter), and finally exposed to gold vapors in the evaporator. Different gold thicknesses (2 nm, 4 nm, 6 nm, and 8 nm, respectively) were evaporated on squared glass coverslips at 1 × 10^−5^ Torr pressure at 25 °C using an evaporation rate of 0.03 nm/s. The resulting gold-coated glasses (4 sets of 12 coverslips/set) were systematically heated on a hot plate preheated to three different temperatures (350 °C, 450 °C, and 550 °C) for different time periods (1, 3, 6, and 9 h, respectively) (Figure 1).

After the annealing procedure, the coverslips underwent an additional cleaning process according to the procedure describe by Jia et al. [6], which involves washing with 70% ethanol in an ultrasonic bath at 30 °C for 20 min, rinsing with sterile water, and further washing in an ultrasonic bath with sterile water for 10 min at 30 °C. Then, the coverslips were allowed to dry in oven at 50 °C for 20 min. After that, the annealed glass coverslips were biofunctionalized by adding 10 μL of thiol-DNA at 10 ng/μL in 1xSSPE at 4 °C overnight. The coverslips were subsequently washed with 1.5 mL of sterile water and dried over the biohood. The thiol-DNA was previously treated with a buffer solution containing 10 mM Tris(2-CarboxyEthyl)Phosphine hydrochloride) (TCEP) and 3 M sodium acetate in order to release the thiol group.

### 2.4. SERS Measurements on Coverslips

Different BPE concentrations were tested (10^−3^, 10^−5^, 10^−7^, 10^−9^, and 10^−12^ M) by deposing tiny drops of 2 μL on gold coverslips. In order to increase the sensitivity of the SERS experiments, different combinations of spectral acquisition time and laser filtering were used: for 10^−3^ M and 10^−5^ M, the acquisition time was 10 s using the D0 filter, whereas for the lower concentrations, a D0.3 filter was used to avoid the background noise due to the longer excitation time necessary for comparable spectra acquisition. An acquisition time of 30–120 s was studied. The stability of SERS spectra over five weeks for gold nanostrutured coverslips modified with thiol-DNA probe with an acquisition time of 10–30 s and using a D0.3 filter is also reported.

## 3. Results and Discussion

### 3.1. SEM Characterization

**Figure 2:**
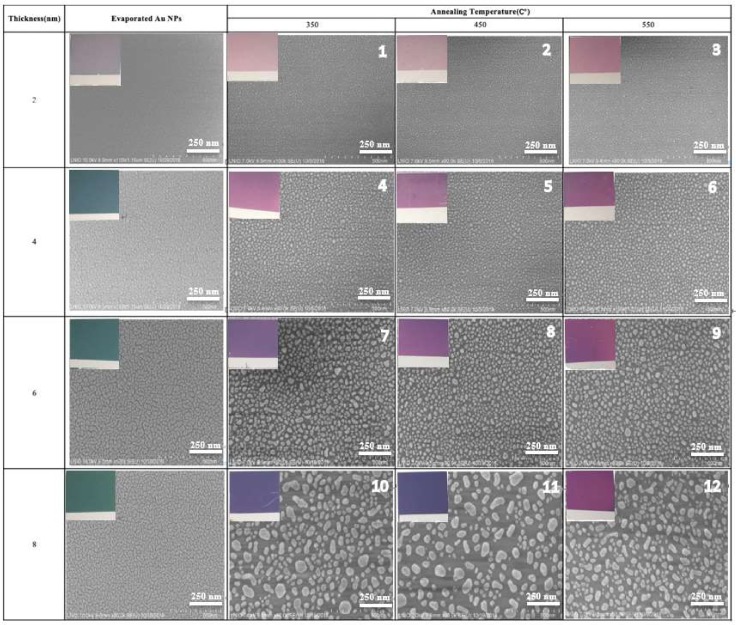
SEM images of square glass coverslips gold coated (2 nm, 4 nm, 6 nm, and 8 nm) after 3 h at different temperatures (350 °C, 450 °C, and 550 °C). AuNPs: gold nanoparticles.

It is well known that the SERS properties of gold nanostructures are strongly influenced by the size, distribution, and spacing between particles [8]. In the preparation of the AuNP process, including cleaning, evaporation, and annealing protocol, the morphology of the substrate can be modulated by controlling different experimental conditions, such as the Millipore and Decon 90 distilled water ratio, gold film thickness, evaporation pressure, evaporation rate, annealing time, and annealing temperature. In our experiments, three parameters—the thickness of the evaporated gold film, the annealing temperature, and the annealing time—played an important role in the morphology of the gold nanoparticles and SERS properties. Thus, SEM studies were conducted for four different gold film thicknesses (2 nm, 4 nm, 6 nm, and 8 nm, respectively) at three different annealing temperatures (350 °C, 450 °C, and 550 °C) and for four different annealing times (1, 3, 6, and 9 h) (Figure 2).

#### 3.1.1. Influence of the Annealing Temperature on the Formation of Gold Nanoparticles

Evaporated gold films of 2 nm, 4 nm, 6 nm, and 8 nm on coverslips showed different colors, from light blue (2 nm Au) to blue (4 nm Au), to light green (6 nm Au), or to darker green (8 nm Au). These colors changed significantly for each gold thickness after 3 h of annealing at different temperatures. The highest temperature produced a violet color for the 2 nm gold film, whereas for the 4 nm, 6 nm, and 8 nm films, the color appeared from light violet to dark purple, respectively (Figure 1). SEM images of the evaporated samples and the annealed samples are shown in Figure 2. The size of the gold nanoparticles increased with the increase of the thickness of the film (2 nm, 4 nm, 6 nm, and 8 nm), which corresponded to the color variation before and after annealing at different temperatures.

**Figure 3:**
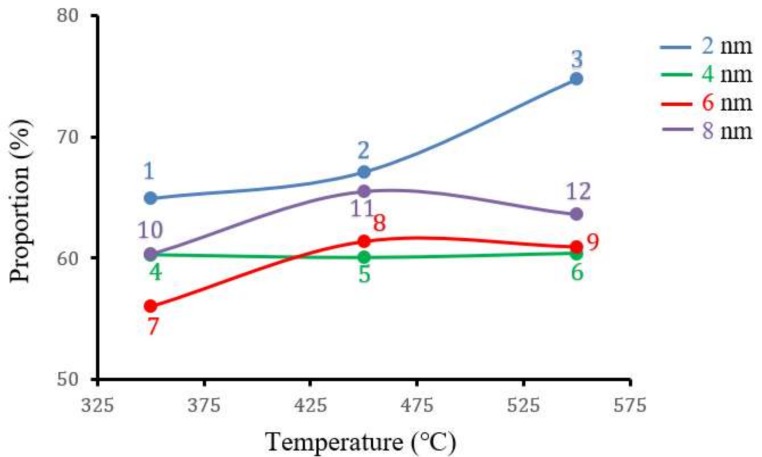
The proportion of background for the annealed square glass coverslips gold coated (2 nm, 4 nm, 6 nm, and 8 nm, respectively) after exposure at three different temperatures (350 °C, 450 °C, and 550 °C). These numbers are also used in Figure 2 to indicate the SEM image for every substrate.

For glass coverslips coated with 2 nm Au, the interparticle distances, or proportion of background (PB), increased when temperatures rose from 350 °C to 550 °C (Figure 3). On the contrary, for samples coated with 6 nm and 8 nm Au, the PB values at 550 °C ranged from 60.94% to 63.63%, while at 450 °C, the PB values were 61.39% and 65.53%, respectively. Interestingly, the PB values for the 4 nm Au sample showed no great variation when annealed at 350 °C (60.29%), 450 °C (60.07%), or 550 °C (60.41%).

In conclusion, the temperature definitively influenced the sizes and shapes of the gold nanoparticles and the interparticle distances, with respect to the gold thickness evaporated on the coverslips.

#### 3.1.2. Influence of Gold Thickness on Coverslips on Nanoparticle Distribution

**Figure 4:**
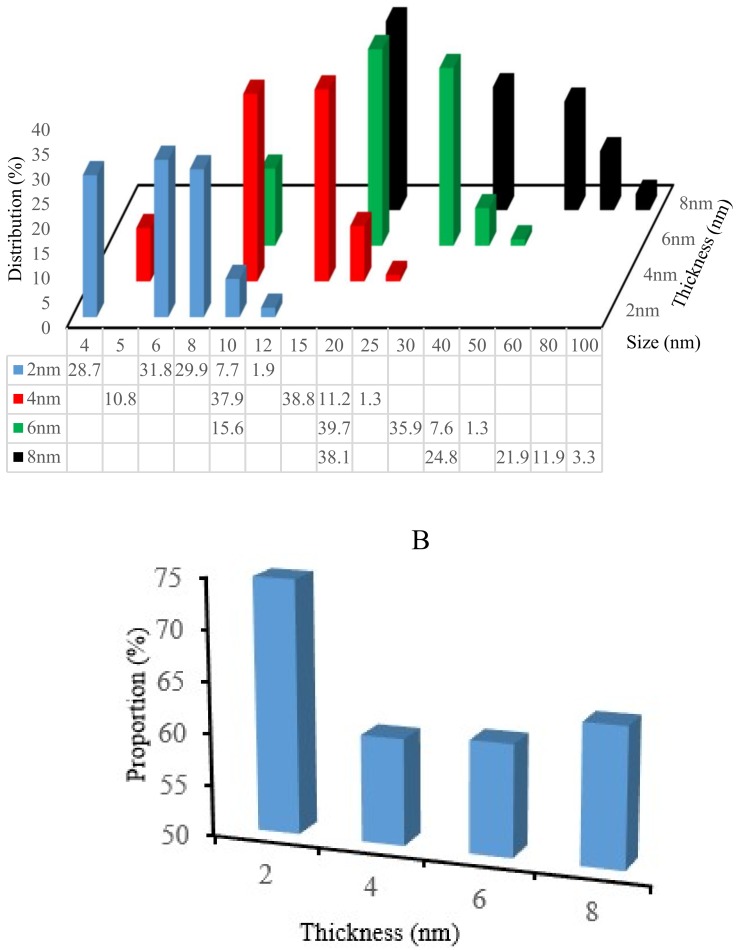
The size distribution of AuNPs on coverslips (**A**) and the proportion of background for different gold thicknesses (2 nm, 4 nm, 6 nm, and 8 nm) after annealing at 550 °C for 3 h (**B**).

As robust and stable SERS platforms, square coverslips coated with gold of 2 nm, 4 nm, 6 nm, and 8 nm were proposed and annealed at 550 °C for 3 h. For these samples, the particle size distribution, and the proportion of background are reported in Figure 4A,B.

Figure 4A shows that by increasing the thickness of gold, the size of the AuNP nanoparticles and their distribution percentage increase. Similarly, the size of nanoparticles affects the interdistance between particles. Thus, after the annealing of 2 nm Au film on the glass, the AuNPs ranged mainly from 6 (size distribution 31.8%) to 8 nm (29.9%), whereas for 4 nm Au, the nanoparticles ranged from 10 (37.9%) to 15 nm (38.8%). For 6 nm Au, the AuNPs ranged from 20 (39.7%) to 30 nm (35.9%), and finally, for 8 nm Au, the AuNPs ranged from 20 (38.1%) to 40 nm (24.8%).

On the other hand, it was found that the proportion of background for the coverslips coated with four different gold thicknesses (Figure 4B) was the smallest for 4 nm Au (60.41%) and the highest for 2 nm Au (74.77%). Additionally, for coverslips coated with 6 nm and 8 nm Au, the background was 60.94% and 63.63%, respectively.

In the current SERS studies, the 4 nm Au coated coverslips after 550 °C showed the largest nanoparticle surface coverage and the lowest interparticle distances compared with the other tested thicknesses (2 nm, 6 nm, and 8 nm, respectively).

#### 3.1.3. Influence of Annealing Time on the Nanostructuration of Coverslips

**Figure 5:**
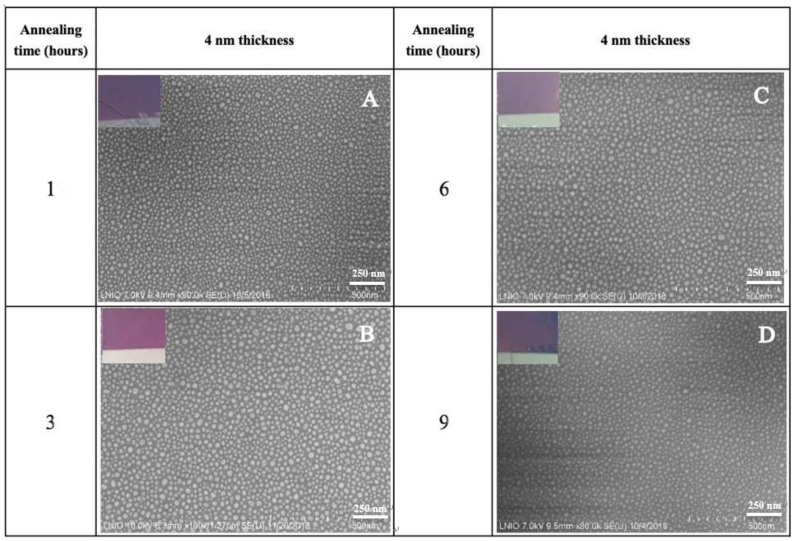
SEM images of AuNPs on square glass coverslips coated with 4 nm and annealed for different time periods (**A**) 1 h, (**B**) 3 h, (**C**) 6 h, and (**D**) 9 h at 550 °C.

**Figure 6:**
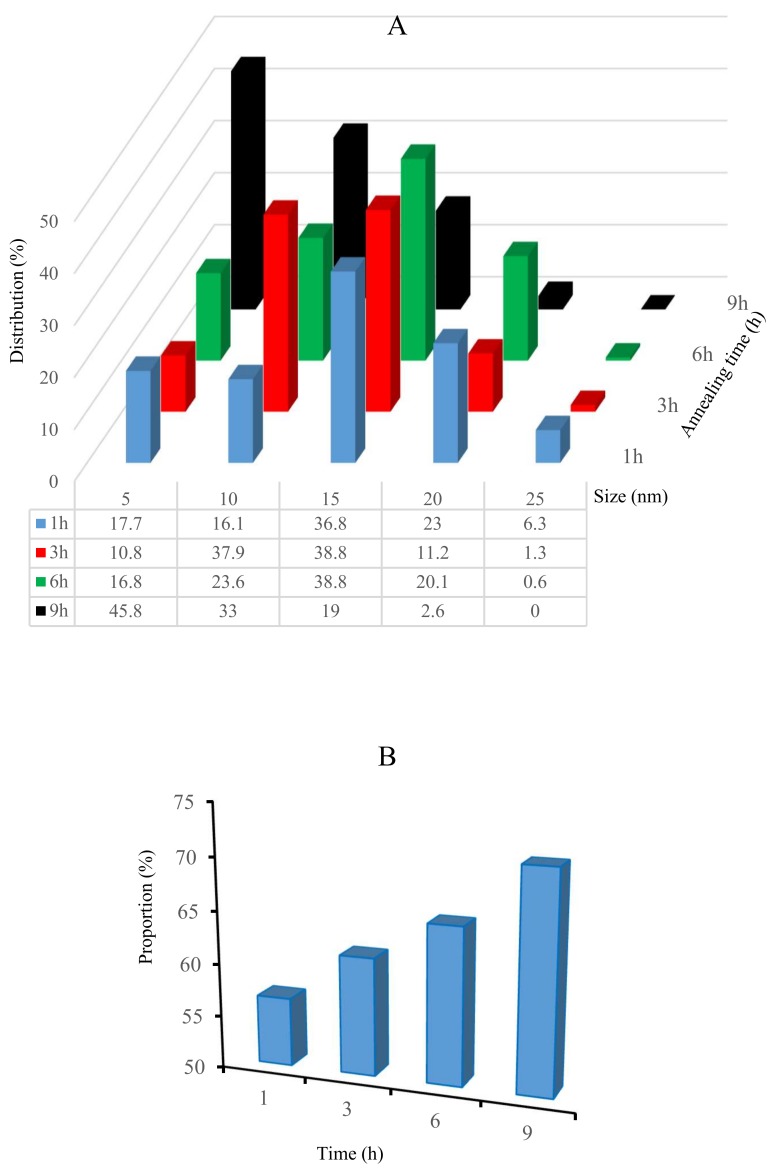
Analysis of AuNPs based on SEM images showing the size distribution of gold nanoparticles on annealed coverslips (**A**) and the proportion of background after annealing the 4 nm gold coated coverslips for different time periods (1, 3, 6, and 9 h) at 550 °C (**B**).

In order to prepare large-scale gold nanoparticles with stable optical characteristics, the effect of 4 nm Au evaporated and annealed at 550 °C at different annealing times (1, 3, 6, and 9 h) is illustrated in Figure 5. On other hand, the nanoparticle size distribution and the proportion of background for SEM images (Figure 2) are analyzed using the public domain ImageJ software platform, developed at National Institutes of Health (Figure 6).

Experimentally, the coverslips heated for 1 h at 550 °C formed nanoparticles in the range of 15–20 nm (36.8–23%). Similar sample evolution was obtained for glasses after 6 h at the same temperature when the AuNPs ranged from 10 (23.6%) to 15 nm (38.8%), while the AuNP size after 3 h displayed a uniform distribution from 10 (37.9%) to 15 nm (38.8%) compared with the others, corresponding to the SEM image (Figure 5).

The coverslips annealed for 9 h showed a high distribution at 5–10 nm (45.8–19.0%) (see Figure 6A). However, as shown in Figure 6B, the proportion of background increased following the evaporated gold film thickness, becoming thicker over time: 9 h (70.72%) > 6 h (64.87%) > 3 h (61.19%) >1 h (56.51%). In detail, even though the sample annealed for 9 h had a very high distribution of 45.8% at 5 nm, the largest proportion of the background of the sample was 70.72%, which corresponded to the coverage of the smallest area of the 9 h sample. On the other hand, the samples annealed for 1 h and 3 h had lower proportions compared with the samples annealed for 6 h and 9 h. The lower proportion of background of the larger surface coverage was obtained, and considering the uniform distribution of the nanoparticles and better surface coverage, we used the samples annealed at 550 °C for 3 h.

### 3.2. SERS Characterization

The SERS tests were initially performed on classical microscope glass slide supports modified with 4 nm gold film and annealed at 550 °C in the oven for 8 h according to the procedure described by Jia et al. [6]. These substrates proved to be inappropriate for SERS measurements, because the glass slide produced strong fluorescence interferences that abnormally altered the optical signals. Therefore, several solid supports are here proposed for SERS investigations: plastic petri dishes, glass coverslips, plastic pipettes, Eppendorf tubes, plastic cuvettes, and quartz crystals microbalance (QCM) (Supplementary Materials, Figure S1). The SERS measurements show that the best solid supports were the ultrafine glass coverslips for further gold nanostructuration due to the absence of fluorescence interferences. Then, the SEM morphology also confirmed the evolution of SERS signals for annealed gold coated coverslips at 350 °C, 450 °C, and 550 °C for 3 h (Figure S2A–C). Among the different spectroscopic investigations (Figure S3), it was found that the best SERS substrate is the ultrafine square glass coverslip coated with 4 nm Au (Figure S3B) annealed at 550 °C for 3 h (Figure S2C), due to the absence of background SERS peaks that could mask the presence of specific SERS peaks produced by (bio)molecules once immobilized on nanoparticles.

#### 3.2.1. SERS Spectrum of BPE Molecule on 4 nm Gold-Annealed Coverslip

**Figure 7:**
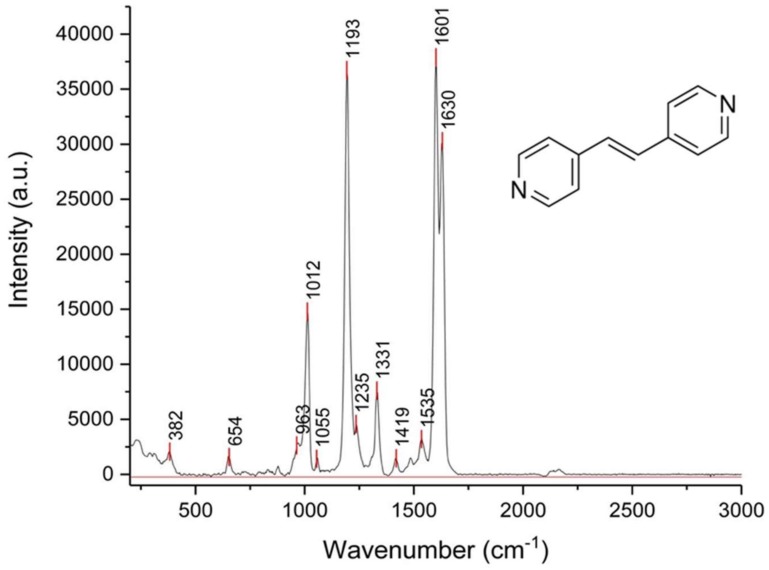
Surface enhanced Raman spectroscopy (SERS) spectrum of the 1,2-bis-(4-pyridyl)-ethene (BPE) (1 mM) on annealed gold nanostructured coverslip (4 nm Au, 550 °C for 3 h on a hot plate), after three times of acquisition of 10 s and using a D0 filter.

The SERS tests were carried out in the presence of a model molecule, 1,2-bis-(4-pyridyl)-ethene, which has interesting bonds and atoms giving good SERS spectra when deposited on annealed gold coated coverslips, as demonstrated in Figure 7.

As reported, the main peaks at 1601 and 1630 cm^−1^ correspond to the C–N stretching mode in the pyridyl ring and the BPE vinyl group vibration, respectively [9], while the peaks at 1193 and 1235 cm^−1^ refer to the ring breathing mode of pyridine and the vibrational movement of the nitrogen atom in pyridyl, respectively. In the present work, the peak at 1012 cm^−1^ can be attributed to the chemical absorption of BPE molecules onto AuNPs on coverslips.

#### 3.2.2. SERS Spectra of Different BPE Concentrations

**Figure 8:**
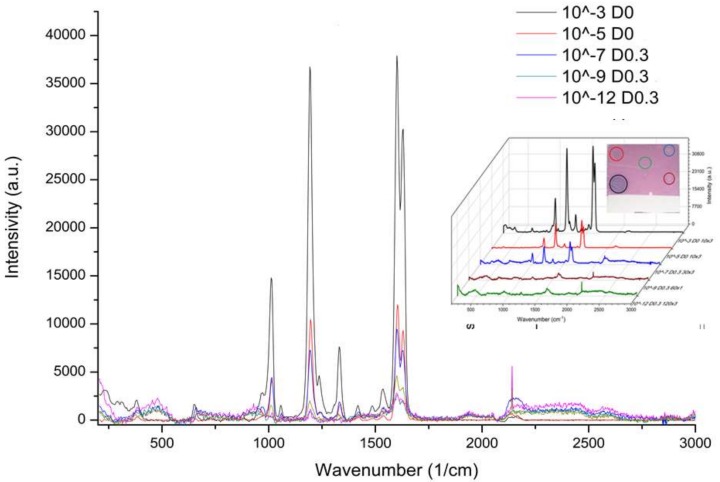
SERS spectra of BPE molecules of different concentrations (10^−3^, 10^−5^, 10^−7^, 10^−9^, and 10^−12^ M) using 4 nm gold-coated coverslips annealed at 550 °C for 3 h on a hot plate. Inset-photo of a coverslip after the deposition of five different BPE concentrations.

SERS signals were recorded and compared for different BPE concentrations deposited on gold-annealed coverslips. These SERS measurements confirmed that the best conditions for the detection of very low BPE concentrations are as follows: 4 nm Au on glass heated at 550 °C for 3 h. As is shown in Figure 8, gold nanostructured coverslips made possible the SERS detection of the lower concentration of the BPE molecule at 10^−12^ M.

**Figure 9:**
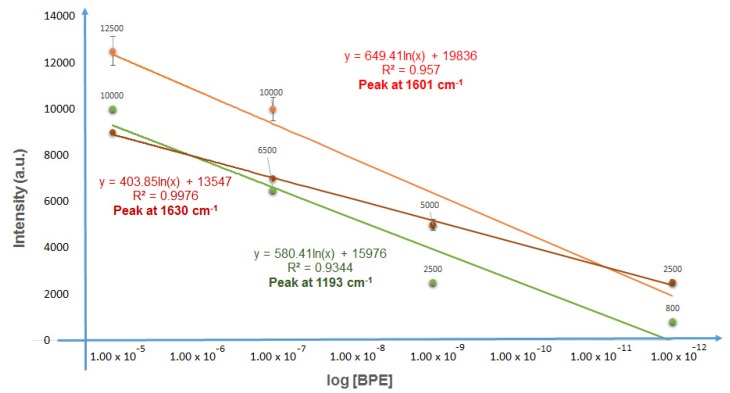
SERS intensity of three wavenumber main peaks (1193 cm^−1^, 1630 cm^−1^, and 1601 cm^−1^) as a function of BPE concentration.

Figure 9 depicts the intensity variation of the three main SERS peaks (1193 cm^−1^, 1630 cm^−1^, and 1601 cm^−1^) versus the decimal logarithmic of four BPE concentrations. Whatever the wavenumber, the intensity variation exhibited an autonomous decay when the concentration of BPE decreased. A linear fit was used to model the experimental SERS measurements. A pronounced linear behavior was observed for the 1630 wavenumber, for which a linear regression coefficient of 0.9976 was calculated. The values of the modeled slopes that represent the sensitivity were of the same order of magnitude.

The enhancement factor (EF) was calculated using the equation EF = (I_SERS_/I_R_) × (N_R_/N_SERS_) and was found to be equal to 2.71 × 10^7^. I_SERS_ represents the intensity of the 1630 cm^−1^ BPE band, measured for 10^−5^ M concentration, while I_R_ represents the intensity of the Raman band for 10^−3^ M on reference glass. N_SERS_ and N_R_ represent the number of molecules formed as a layer of 10 nm thickness under the laser spot and the number of the BPE molecules in the focal volume. The values of I_R_ and N_R_ are the same as those reported in [28]. Gold nanoparticles covered 40% of the surface under the spot. By using the same calculation method, N_SERS_ was found to be equal to 125 molecules for a surface spot laser of 5.2 µm^2^. In the case of 10^−5^ M BPE content, an I_SERS_ value of 9000 was measured.

#### 3.2.3. SERS Signal Stability of Thiol-DNA Deposited on Gold-Annealed Coverslip Substrate

**Figure 10:**
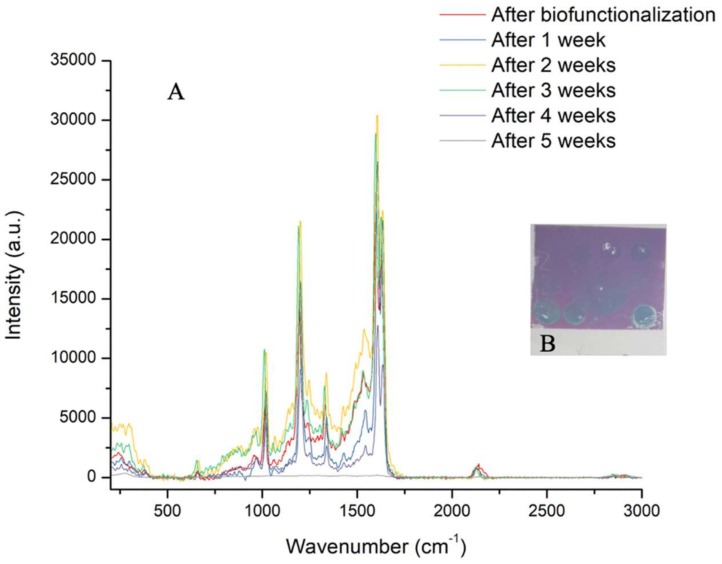
Evolution of SERS intensity of the thiol-DNA probe on annealed gold-coated coverslip over five weeks and obtained after three acquisition times, showing a 10 s spectra with a D0.3 filter (**A**). Photo of the sample after five weeks (**B**).

A thiol-DNA biofunctionalized gold-annealed coverslip was tested over five weeks to evaluate the substrate’s SERS signal stability (Figure 10). Interestingly, the intensity of SERS increased after two weeks and decreased after four weeks. This confirms that the nanostructuration of the coverslip was stable for more than a month. The stability tests were stopped after five weeks, because the thiol-DNA probe on AuNPs presented a strong attenuation of the SERS intensity.

## 4. Conclusions

Large-scale, annealed, gold nanostructures were fabricated for the first time on ultrafine glass coverslips. Several parameters have been optimized to conclude that 4 nm gold-coated coverslip heated at 550 °C on a hot plate for 3 h had the greater sensitivity of the SERS spectrum to different BPE concentrations. By using the newly SERS annealed coverslips platforms it was possible to detect a BPE concentration of 10^−12^ M. Moreover, the stability of SERS spectra intensity over five weeks of a thiol-DNA probe (10 ng/µL) was also monitored. On the basis of these results, annealed gold coverslips can be considered as ideal substrates in the construction of ultrasensitive SERS nanobiosensors.

## Supplementary Materials

The following are available online at https://www.mdpi.com/2079-6374/9/2/53/s1, Figure S1: SERS spectra of various naked solid supports: plastic petri dish, glass coverslip, plastic pipette, Eppendorf tube, plastic cuvette and quartz QCM crystal, Figure S2: SERS signals of naked annealed gold films (2, 4, 6 and 8 nm) on coverslips after 3 h, Figure S3: SERS signals of naked annealed gold films on coverslips at 550 °C for different periods.
