# Supplementary material for: Robust SERS Platforms Based on Annealed Gold Nanostructures Formed on Ultrafine Glass Substrates for Various (Bio)Applications
Source: Biosensors (Basel). 2019 Apr 10;9(2):53. doi: 10.3390/bios9020053 (PMC6627616; doi:10.3390/bios9020053)
Supplement: Supplementary file 1 [file biosensors-09-00053-s001.pdf]

biosensors

MDPI

Supplementary Material

Robust SERS Platforms Based on Annealed Gold Nanostructures Formed on Ultrafine Glass Substrates for Various (Bio)Applications

Lan Zhou [1], Simone Poggesi [1,2], Giuliocesare Casari Bariani [1,2], Rakesh Mittapalli [1], Pierre-Michel Adam [1], Marisa Manzano [2] and Rodica Elena Ionescu [1,*]

[1] Light, Nanomaterials and Nanotechnology (L2N), FRE-CNRS 2019, Institute Charles Delaunay (ICD), University of Technology of Troyes, 12 Rue Marie Curie CS 42060, 10004 Troyes CEDEX, France; lan.zhou@utt.fr (L.Z.); poggesi.simone@spes.uniud.it (S.P.); casaribariani.giuliocesare@spes.uniud.it (G.C.B.); rakesh.mittapalli@utt.fr (R.M.); pierre_michel.adam@utt.fr (P.-M.A.)

[2] Dipartimento di Scienze Agroalimentari, Ambientali e Animali (DI4A), Università degli Studi di Udine, Via Sondrio 2/A, 33100 Udine, Italy; marisa.manzano@uniud.it

* Correspondance: elena_rodica.ionescu@utt.fr; Tel.: +33-3-2575-9728; Fax: +33-3-2571-8456

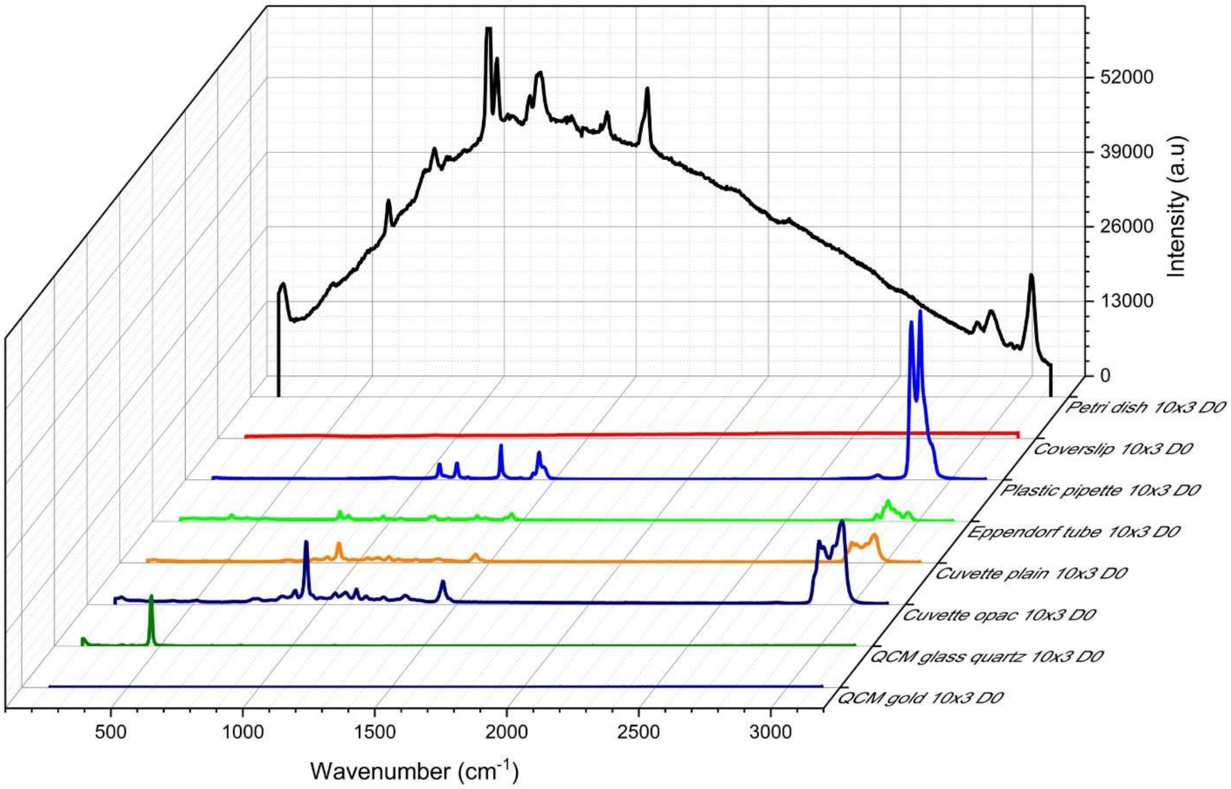

Figure S1. SERS spectra of various naked solid supports: plastic petri dish, glass coverslip, plastic pipette, Eppendorf tube, plastic cuvette and quartz QCM crystal.

A. 350 °C for 3 h

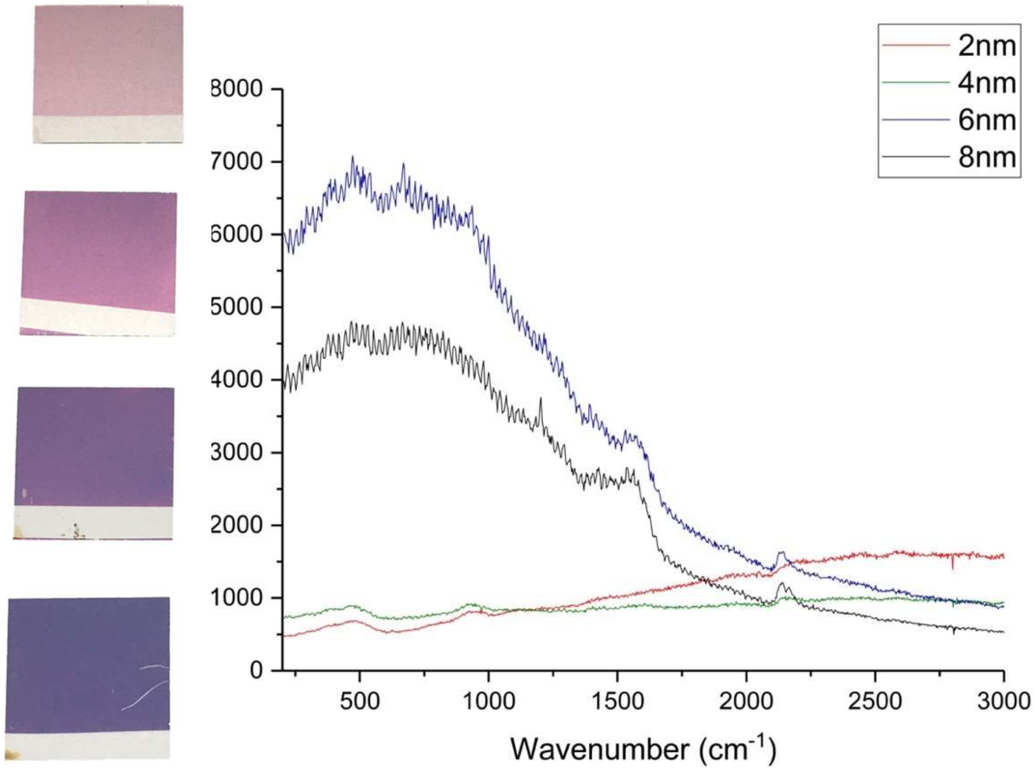

B. 450 °C for 3 h.

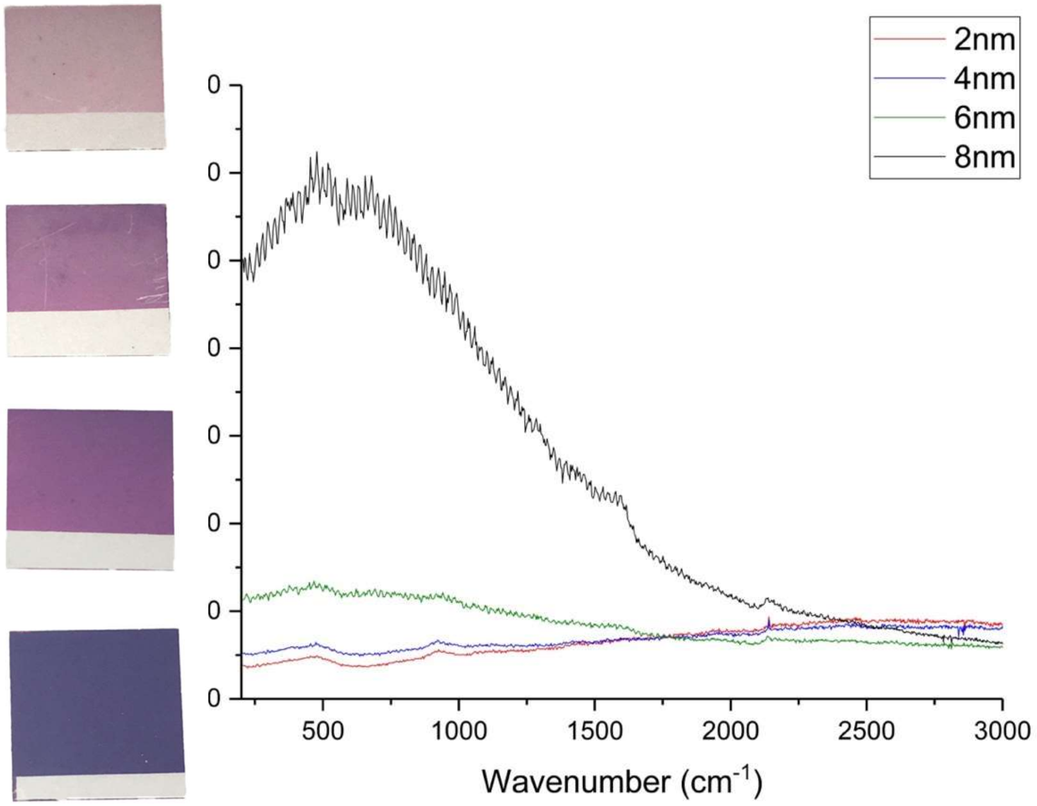

C. 550 °C for 3 h

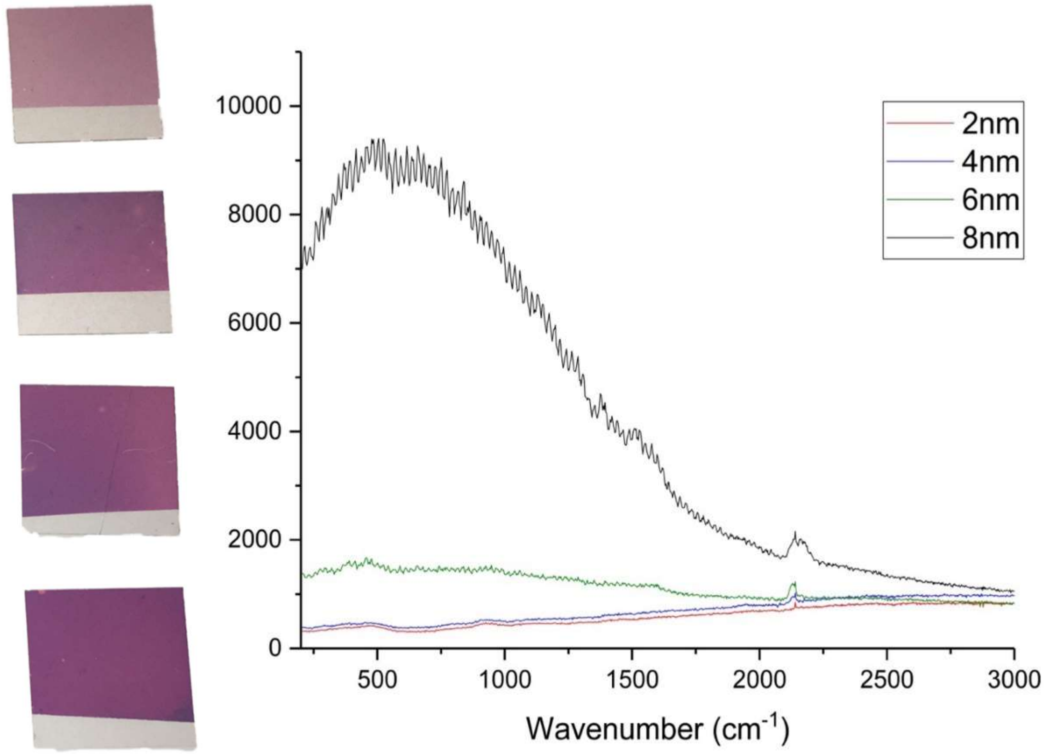

Figure S2. SERS signals of naked annealed gold films (2, 4, 6 and 8 nm) on coverslips after 3h.

A. 2 nm Au

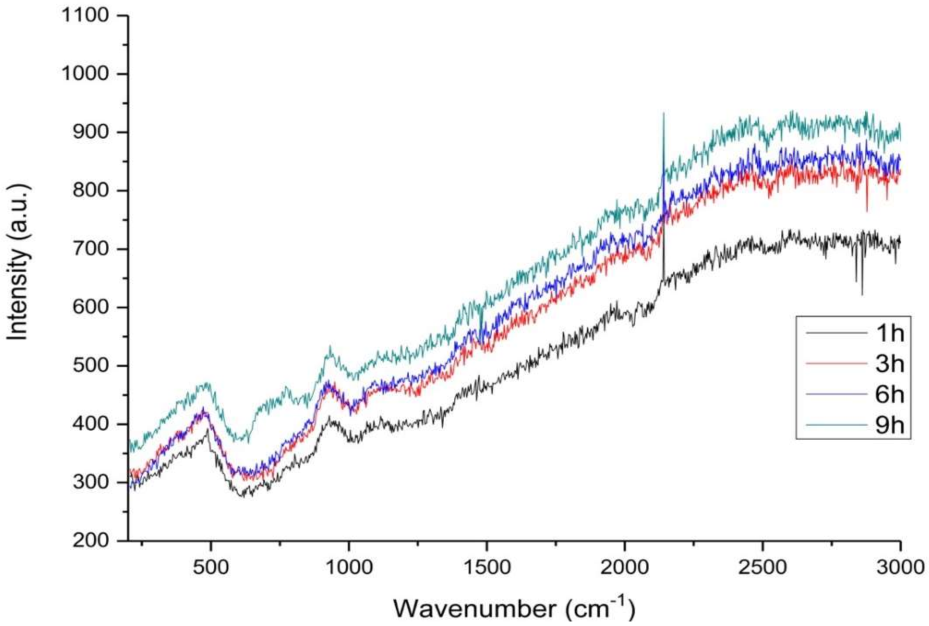

B. 4 nm

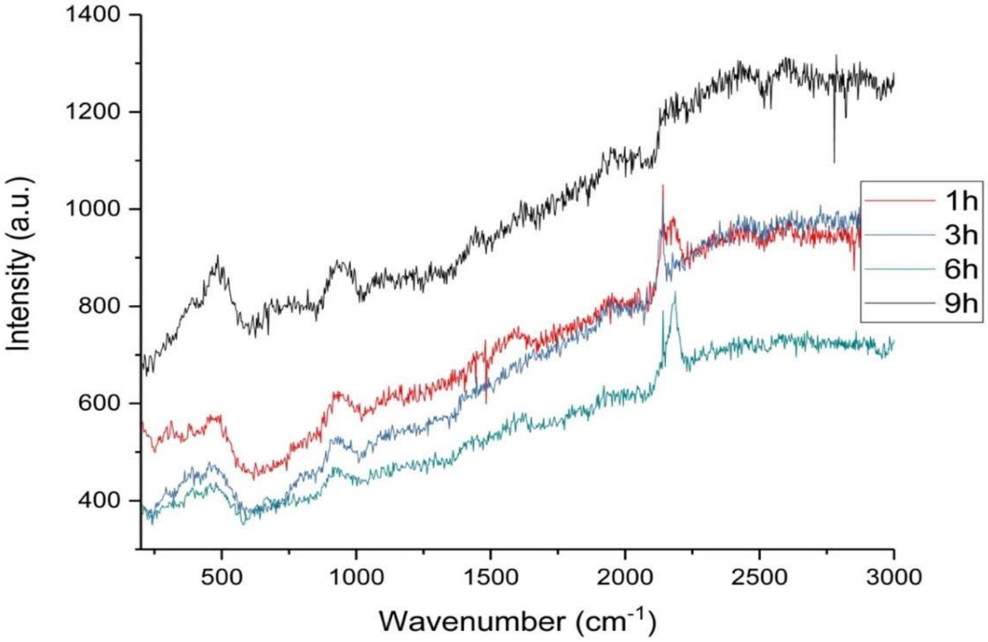
1400
1200
1000
800
600
400
500
1000
1500
2000
2500
3000
Intensity (a.u.)
Wavenumber (cm-1)
1h
3h
6h
9h

C. 6 nm

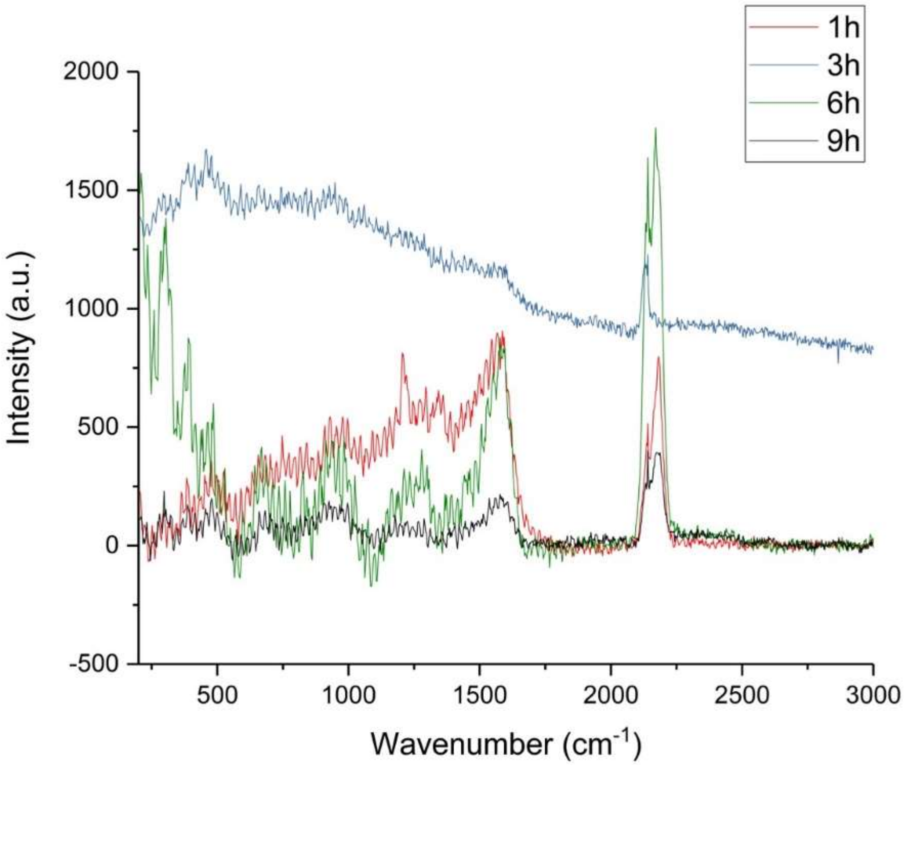
1h
3h
6h
9h
2000
1500
1000
500
0
-500
500
1000
1500
2000
2500
3000
Intensity (a.u.)
Wavenumber (cm-1)

D. 8 nm

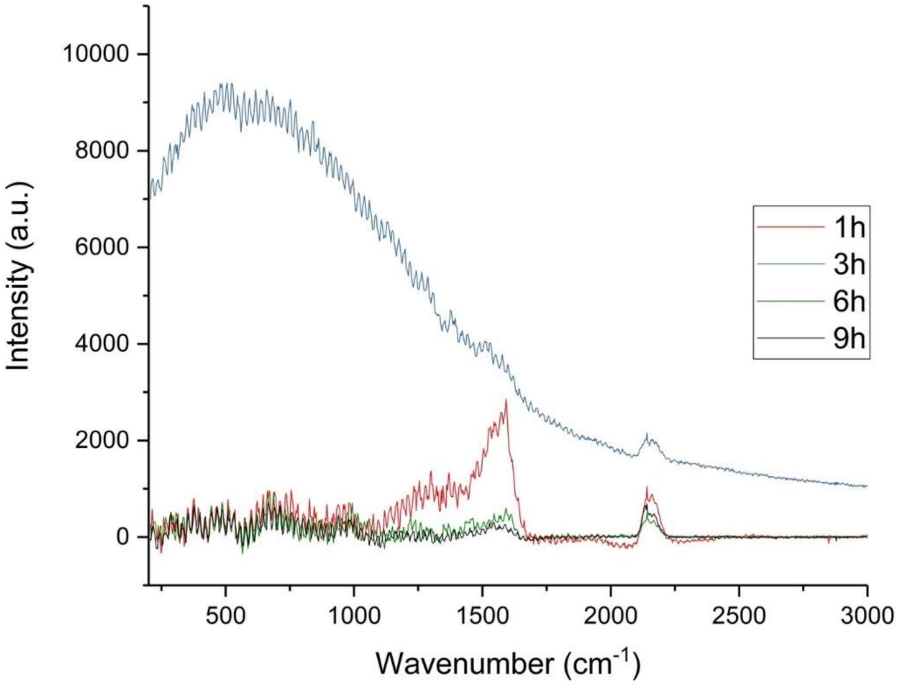

Figure S3. SERS signals of naked annealed gold films on coverslips at 550 °C for different periods.
